# Association between Vitamin D Status and Secondary Infections in Patients with Severe COVID-19 Admitted in the Intensive Care Unit of a Tertiary-Level Hospital in Turkey

**DOI:** 10.3390/diagnostics13010059

**Published:** 2022-12-26

**Authors:** Lutfiye Karcioglu Batur, Suna Koç

**Affiliations:** 1 Department of Molecular Biology and Genetics, Faculty of Engineering and Natural Sciences, Biruni University, Istanbul 34010, Turkey; 2 Department of Anesthesiology and Reanimation, Medical Faculty, Biruni University, Istanbul 34010, Turkey

**Keywords:** vitamin D status, COVID-19, Intensive Care Unit, secondary infections

## Abstract

There are several studies showing that the vitamin D status can determine risk of COVID-19 infections, severity and mortality from coronavirus disease 2019 (COVID-19). However, the association between vitamin D (25(OH)D) and secondary infections in the prognosis of COVID-19 patients has not been reported yet. The aim was to investigate whether the vitamin D status affects the rates of secondary infections in patients with severe COVID-19 hospitalized in the intensive care unit (ICU) of a tertiary-level hospital in Turkey. The data of 194 patients with diagnosis of severe COVID-19 who were admitted to the ICU from March 2020 to June 2021 and older than 18 years were evaluated in this retrospective study. The patients were divided into two groups according to total serum 25(OH)D level as normal group (≥20 ng/mL) and low group (<20 ng/mL). The 25(OH)D level was low in 118 (60.8%) and normal in 76 (39.2%) patients. The mean age of the low group was significantly higher than that of the normal group (67.02 ± 14.47 vs. 61.70 ± 14.38; *p* = 0.013). The systolic and diastolic blood pressure as well as the Glasgow coma scale score of the low group were significantly lower than that of the normal group (*p* = 0.004, 0.002 and 0.001, respectively). The intubation rate and APACHE (Acute Physiology and Chronic Health Evaluation) score of the low group was significantly higher than that of the normal group (*p* = 0.001). The platelets number and blood pH decreased, and the neutrophil/lymphocyte ratio, procalcitonin, lactate, urea, creatinine and lactate dehydrogenase concentrations increased significantly in the low group (*p* < 0.05). The mortality rate was 79.7% in the low group and 22.4% in the normal group (*p* < 0.001). Microbiological growth was observed in 68.6% of the normal group and 52.6% of the normal group (*p* = 0.025). The number of cultures with resistant bacteria was significantly higher in the low group (25.9%) than that in the normal group (17.5%) (*p* = 0.035). The severe COVID-19 patients hospitalized with vitamin D deficiency may have increased risks of poor prognosis and mortality due to secondary infections in the ICU.

## 1. Introduction

Vitamin D has been previously reported to have a functional role in preventing and treating the viral infections [1,2] even against novel COVID-19 [3]. This role of vitamin D was supported by the fact that sufficient blood vitamin D levels play an effective role in immune system functioning, which can help in a satisfactory cellular response and in protecting against the severity of infections caused by microorganisms (Ali 2020). Vitamin D deficiency (25(OH)D < 20 ng/mL) has been correlated with severe COVID-19 [4]. resulting in arguments on the benefits of supplementation of this vitamin when treating the illness caused by SARS-CoV-2 (Severe Acute Respiratory Syndrome Coronavirus 2) [5]. However, there are controversial reports for the contribution of this deficiency in the increasing risks of susceptibility to COVID-19 infections [2,6].

A number of reports have shown that the patients’ vitamin D levels are related to controlling the progression of COVID-19 and the risk of mortality due to the infection; however, it is not the only factor related to the prognosis of disease [2,7,8]. Other factors included the previous comorbidities, gender, nutritional status and especially age [9]. Since reduced blood 25(OH)D levels are more prevalent in the elderly portion of the population, vitamin D deficiency may worsen the severity and prognosis of elder COVID-19 patients [10,11]. In addition to the severity of the disease, a sufficient vitamin D status can modulate the C-reactive protein (CRP) levels, which is an inflammatory marker increased in infections, and it can suppress the inflammatory cytokine storm caused by COVID-19, suggesting an immunomodulatory function in viral infections [12]. However, there are a limited number of data showing a possible correlation between vitamin D status and the risk of secondary infections in the prognosis and mortality of patients with severe COVID-19 [13,14]. The aim of the present study was to investigate whether vitamin D status affects the rates of secondary infections in patients with severe COVID-19 hospitalized in the intensive care unit (ICU) of a tertiary-level hospital in Turkey.

## 2. Materials and Methods

### 2.1. Study Design

This retrospective cross-sectional cohort study recruited a total of 194 patients who were diagnosed with severe COVID-19 and followed up in the ICU of a single-center hospital between March 2020 and June 2021. Age-matched 30 patients followed up for non-COVID-19 reasons in the ICU were selected as the control group for comparison of the vitamin D levels. All demographics, comorbidities, initial vital signs, clinical and laboratory findings in admission in ICU, microbiological findings, clinical course, hospitalization and duration of weaning from mechanical ventilation, and mortality rates were recorded from the electronic system of our hospital. The study protocol was approved by the Non-interventional Research Ethics Committee of Biruni University (Date: 25 November 2021; Number: 2021/62-13) after an official permission was received from the Turkish Ministry of Health, Scientific Research Committee for COVID-19. Written informed consent was collected from all participants or their guardians before data selection. The study was conducted in accordance with the principles of Declaration of Helsinki and World Health Organization.

### 2.2. Inclusion and Exclusion Criteria

The inclusion criteria were a positive test confirmed for SARS-CoV-2 via RT-PCR (real-time reverse transcription-polymerase chain reaction), being between 18 and 90 years old and having been hospitalized in ICU at least three days. The severity of COVID-19 was classified as the moderate, severe, mild and critical disease according to the National Institute of Health [15]. Patients who have a ratio of arterial partial pressure of oxygen to fraction of inspired oxygen (PaO_2_/FiO_2_) < 300 mm Hg, SpO_2_ < 94% on room air at sea level, a respiratory rate >30 breaths/min, or lung infiltrates > 50% were accepted as severe illness and selected for the study. Patients diagnosed with mild to moderate COVID-19, patients hospitalized in services for COVID-19 or who had been in the ICU less than three days were excluded from the study.

The patients were divided into two groups according to the total vitamin D status as the normal group had a serum level of total 25(OH)D ≥ 20 ng/mL, and the low group had a serum level of total 25(OH)D < 20 ng/mL according to the literature [16].

### 2.3. Laboratory Tests

All the tests were performed in the clinical laboratory of Biruni University under the standard practices determined by the guidelines of Turkish Ministry of Health. All hormone tests including total 25(OH)D and biochemical analysis were performed by ARCHITECT plus ci4100 device (Abbott Diagnostics, IL, USA).

Microbiological analysis was performed by routine culturing techniques. During the culture antibiograms running, if there was a growth in the culture after the first 24 h, a bacterial identification colorimetric method was applied by a compact device for bacterial identification. The antibiogram cards were used for the antibiotics. The results were collected on the 3rd day of culturing.

### 2.4. Treatments and Follow-Up

Hemodiafiltration (Continuous Venovenous Hemofiltration: CVVH) was performed by a hemodialysis device (Fresenius-multiFiltrate Ci-Ca®) based on the heparin or citrate anticoagulation in patients with acute renal failure for a 24-h period.

Hemoperfusion was also performed by a cartridge-directed hemadsorption device (HA330 Sepsis Adsorption Column, Jafron Biomedical Co., Ltd., China) in the patients with sepsis.

CP was used to reduce the effects of the disease and the transmission process in COVID-19 patients with severe progression. On a voluntary basis, the plasma obtained from individuals who have had COVID-19 infection by a blood donation process was separated by an apheresis device, and the other components was given back to the blood donor. The routine standard tests were applied to the plasma collected from patients who have had the disease and recovered but still have antibodies and proteins formed against the virus. Depending on the severity of the patient’s clinical condition, this treatment was applied once or as repeated doses.

The medicines used for treatments were methylprednisolone, Actemra, Favipravir and hydroxychloroquine. The methylprednisolone (Prednol 1000 mg) was applied as 1 × 1 at a high dose for 3 days; then, the dose was reduced by half every day and applied as 1 × 40 mg after 1 week. The Acemtra was applied as 4–8 mg/kg for 3–5 days depending on the clinical findings. The loading dose of hydroxychloroquine (Plaquenil 200 mg) was applied as 2 × 400 mg and then as 2 × 200 mg for a total of 5 days.

### 2.5. Statistical Analysis

All statistical analysis was performed by using the SPSS (Statistical Package for the Social Sciences) Statistics 22 program (IBM Corp, Armonk, NY, USA). The Kolmogorov–Smirnov test was used to test the normality of variables. Normally distributed quantitative variables were given as mean ± standard deviation, and non-normally distributed quantitative variables were given as median and range. The normally distributed quantitative variables between two groups were compared by Student’s *t* test and non-normally distributed quantitative variables were compared by the Mann–Whitney U test. The qualitative variables were given as frequency and percentages and compared by Chi-square test, Fisher’s exact test and continuity (Yates) correction. The significance level for *p*-value was determined as 0.05.

## 3. Results

Among the patients hospitalized in the ICU between March 2020 and June 2021, 30 patients treated for non-COVID-19 reasons were selected as the control group. The mean age of these non-COVID patients was 62.69 ± 13.9 years, which was not dissimilar from the mean age of COVID-19 patients (64.93 ± 14.63 years (25–93 years); *p* = 0.425). The mean serum 25(OH)D level of non-COVID patients was 29.6 ± 9.97 ng/mL which was significantly higher than those of COVID-19 patients (18.83 ± 12.0 ng/mL; *p* < 0.0001). The total serum 25(OH)D level was low in 118 (60.8%) of severe COVID-19 patients hospitalized in the ICU and normal in 76 (39.2%) patients. The mean age of patients with 25(OH)D level < 20 ng/mL (low group) was significantly higher than those of patients with 25(OH)D level ≥ 20 ng/mL (normal group) (*p* = 0.013). Of the 194, 106 were male (54.6%) and 88 were female (45.4%), and the two groups did not differ in terms of sex distribution (*p* = 0.103). More than half of the patients had hypertension (52.6%), 33.5% had diabetes mellitus, 23.2% had a CAD (coronary artery disease) and 52.1% had other comorbidities. There was no significant difference between groups in terms of distribution of any comorbidity (*p* > 0.05) (Table 1).

The clinical and vital findings of COVID-19 patients at the admission to ICU were compared according to the vitamin D status, and the results are presented in Table 2. The pulse, temperature, respiratory rate, the intubation duration and SOFA (Sequential Organ Failure Assessment) scores did not differ between two groups (*p* > 0.05). However, the mean systolic blood pressure (SAP), diastolic blood pressure (DAP) and the mean Glasgow coma scale (GCS) score were significantly lower in the patients with low serum level of vitamin D compared to those of the normal group (*p* = 0.004, 0.002 and 0.001, respectively). The intubation rate among the patients with low levels of vitamin D was significantly higher than those of the normal group (41.5% vs. 17.1%; *p* = 0.001). The mean APACHE (Acute Physiology and Chronic Health Evaluation) score of the patients with low levels of vitamin D was also significantly higher than those of the normal group (30.57 ± 13.71 vs. 20.73 ± 12.05; *p* = 0.001) (Table 2).

The laboratory findings of COVID-19 patients were compared according to the vitamin D status, which are all presented in Table 3. The number of white blood cells (WBC), hemoglobin (HGB), neutrophils, mean platelet/lymphocyte ratio (PLR), the level of C-reactive protein (CRP), troponin, D-dimer, aminotransferase (AST), alanine aminotransferase (ALT), ferritin and fibrinogen did not differ statistically between two groups (*p* > 0.05). In the same period, only five of the patients in the control group hospitalized in the intensive care unit for any reason other than COVID-19 had 25 (OH) D levels below 20 ng/mL. However, the number of platelets and pH of blood was higher in the patients with low serum level of vitamin D compared to those with normal serum level of vitamin D (*p* = 0.002 and 0.019, respectively). The neutrophil/lymphocyte ratio (NLR), the median concentrations of procalcitonin (PCT), lactate, urea, creatinine and lactate dehydrogenase (LDH) significantly increased in the patients with low serum level of vitamin D compared to those with normal level of vitamin D (*p* < 0.05) (Table 3).

The distribution of patients having treatments for COVID-19 was compared according to the vitamin D status (Table 4). The number of patients given convalescent (immune) plasma (CP), methylprednisolone, Actemra, favipiravir and hydroxychloroquine did not differ statistically between two groups (*p* > 0.05). However, 30.2% of patients in the low group had hemodiafiltration, while four patients in the normal group (5.3%) had this treatment (*p* < 0.001). Overall, 29.3% of patients in the low group had hemoperfusion while 14.5% of patients in the normal group had this treatment (*p* = 0.028) (Table 4). In people receiving hemoperfusion therapy, there is no statistically significant difference between vitamin D levels in terms of mean age and gender distribution (*p* > 0.05). There is no statistically significant difference between vitamin D levels in terms of hospital stay (*p* > 0.05).

The prognostic factors of severe COVID-19 patients including the weaning duration, hospitalization duration and mortality rates were compared according to vitamin D status and presented in Table 5. No significant difference was found for the weaning and hospitalization durations between two groups (*p* = 0.612 and 0.308, respectively). However, a high mortality rate was detected among the patients with low levels of vitamin D (79.7%), while the rate in the normal group was 22.4%, and a significant difference was found between the two groups (*p* < 0.001) (Table 5).

The microbiological findings of COVID-19 patients during the course of disease were compared according to the vitamin D status and presented in Table 6. A microbiological growth was observed in the cultures of 68.6% of patients with low serum levels of vitamin D, whereas it was observed in those of 52.6% of patients with normal levels (*p* = 0.025). The number of cultures with resistant bacteria was significantly higher in the cultures of patients in the low group (25.9%) than those in normal group (17.5%) (*p* = 0.035). However, the microbiological proliferation duration was not statistically different between two groups (*p* = 0.441) (Table 6). Among 121 patients who had microbiological growth in cultures, the mortality rate was higher in the patients with low levels of vitamin D (91.4%) than those of the normal group (37.5%) (*p* < 0.001), but the hospitalization duration in ICU did not differ statistically between the two groups (*p* = 0.368) (Table 7).

## 4. Discussion

The present study indicated that the low serum level of total 25(OH)D is associated with a poor prognosis and increased mortality rates among patients with severe COVID-19 admitted to ICU of a tertiary-level hospital in Turkey, and this association may depend on the secondary microbial infections. The fact that only five out of 30 patients in the control group hospitalized in the ICU for any reason other than COVID-19 had a 25 (OH) D level of <20 ng/mL suggests that a low vitamin D value in COVID-19 patients is an issue that should be emphasized. There are many studies supporting this [2,17]. The present study indicated that the low serum level of total 25(OH)D is associated with a poor prognosis and increased mortality rates among patients with severe COVID-19 admitted to the ICU of a tertiary-level hospital in Turkey, and this association may depend on the secondary microbial infections. In detail, the mean age of patients with vitamin D deficiency was higher than that of patients with normal serum level of vitamin D. Although the vitamin D level changes seasonally, biological materials were collected between March 2020 and June 2021. The vital and clinical findings including SAP, DAP, intubation rate, GCS score and APACHE score varied depending on the vitamin D status. In addition, the laboratory findings including the number of platelets, NLR, PCT, blood pH, the concentrations of lactate, urea, creatinine and LDH impaired in the patients with vitamin D deficiency compared to the patients with normal levels. More patients in the low level of vitamin D group need hemodiafiltration and hemoperfusion compared to the patients with normal levels. What is more to the point, the microbiological growths and the proliferation of resistant bacteria was higher in the cultures of patients with vitamin D deficiency than those of patients with normal levels. The mortality rate of the patients with low serum levels of 25(OH)D was higher than that those of the normal group, and the mortality rate among patients who had microbiological growth in cultures was higher in the low group as well. These results are comparable with the observational studies reporting that serum 25(OH)D levels are inversely correlated with the incidence or severity of COVID-19, and vitamin D deficiency may increase the risk of hospitalization and mortality rates from COVID-19 [2,18].

As a powerful epigenetic regulator, vitamin D affects the expressions of more than 2500 genes [19]. Vitamin D has been demonstrated to affect the immune system and hence modulate the inflammatory status [20,21]. A number of studies have reported that vitamin D activates immune cells to produce antimicrobial peptides (AMPs) such as the distinct molecules known as cathelicidins and defensins, which have roles in an innate immune system [22,23]. In addition to commonly known antimicrobial activities, AMPs have also antiviral effects which inactivate the influenza virus [24]. The antiviral activities of AMPs depend on damaging the envelope proteins by cathelicidin [25]. Therefore, the vitamin D status may reduce the survival and replication of the virus by the induction of cathelicidin and defensins. Since the body’s immune system becomes dysregulated in severe COVID-19, the phenomenon of the immune regulatory role of vitamin D in viral infections by the induction of cathelicidin and defensins may explain the poor prognosis, increased risk of secondary infections and higher mortality rates of the patients with vitamin D deficiency especially among severe cases hospitalized in ICU.

SARS-CoV-2 infection interacts with and downregulates angiotensin-converting enzyme 2 (ACE2), leading to an excessive accumulation of angiotensin II. Cell cultures of human alveolar type II cells with vitamin D have demonstrated that the virus binds to the ACE2 receptor and reduces its activity and, in turn, promotes ACE1 activity, forming more angiotensin II, which increases the severity of COVID-19 [26]. That effect may also be related to the vitamin D binding protein [4]. The vitamin D metabolite calcitriol was shown to increase the expression of ACE2 in the lungs of experimental animals [27], which promotes the binding of the virus and prevents the constriction response of the lung blood vessel in COVID-19, thereby preventing SARS-CoV-2 from entering cells via the ACE2 receptor [28]. This mechanism may explain how severe COVID-19 cases with vitamin D deficiency are prone to secondary infections, tending to a poor prognosis and high risk of mortality due to SARS-CoV-2 infections. In detail, the mean age of patients with vitamin D deficiency was higher than those of patients with normal serum levels of Vitamin D. Although the vitamin D level changes seasonally, biological materials were collected between March 2020 and June 2021. The vital and clinical findings including SAP, DAP, intubation rate, GCS score and APACHE score varied depending on the vitamin D status. In addition, the laboratory findings including the number of platelets, NLR, PCT, blood pH, the concentrations of lactate, urea, creatinine and LDH impaired in the patients with vitamin D deficiency compared to the patients with normal levels. More patients in the low level of vitamin D group need hemodiafiltration and hemoperfusion compared to the patients with normal levels. What is more, the microbiological growths and the proliferation of resistant bacteria were higher in the cultures of patients with vitamin D deficiency than those of patients with normal levels. The mortality rate of the patients with low serum levels of 25(OH)D was higher than that those of the normal group, and the mortality rate among patients who had microbiological growth in cultures was higher in the low group as well. These results are comparable with the observational studies reporting that serum 25(OH)D levels are inversely correlated with the incidence or severity of COVID-19, and vitamin D deficiency may increase the risk of hospitalization and mortality rates from COVID-19 [2,18].

As a powerful epigenetic regulator, vitamin D affects the expressions of more than 2500 genes [19]. Vitamin D has been demonstrated to affect the immune system and hence modulate the inflammatory status [20,21]. A number of studies have reported that vitamin D activates immune cells to produce antimicrobial peptides (AMPs) such as the distinct molecules known as cathelicidins and defensins, which have roles in the innate immune system [22,23]. In addition to commonly known antimicrobial activities, AMPs have also antiviral effects which inactivate the influenza virus [24]. The antiviral activities of AMPs depend on damaging the envelope proteins by cathelicidin [25]. Therefore, the vitamin D status may reduce the survival and replication of the virus by the induction of cathelicidin and defensins. Since the body’s immune system becomes dysregulated in severe COVID-19, the phenomenon on the immune regulatory role of vitamin D in viral infections by induction of cathelicidin and defensins may explain the poor prognosis, increased risk of secondary infections and higher mortality rates of the patients with vitamin D deficiency especially among severe cases hospitalized in the ICU.

SARS-CoV-2 infection interacts with and downregulates angiotensin-converting enzyme 2 (ACE2), leading to an excessive accumulation of angiotensin II. Cell cultures of human alveolar type II cells with vitamin D have demonstrated that the virus binds to the ACE2 receptor and reduces its activity and, in turn, promotes ACE1 activity, forming more angiotensin II, which increases the severity of COVID-19 [26]. That effect may also be related to the vitamin D binding protein [4]. Vitamin D metabolite calcitriol was shown to increase the expression of ACE2 in the lungs of experimental animals [27], which promotes the binding of the virus and prevents the constriction response of the lung blood vessel in COVID-19, thereby preventing SARS-CoV-2 from entering cells via the ACE2 receptor [28]. This mechanism may explain how severe COVID-19 cases with vitamin D deficiency are prone to secondary infections, tending to a poor prognosis and high risk of mortality due to SARS-CoV-2 infections.

Severe infections in COVID-19 may result in a “cytokine storm”, which is manifested as hyperinflammation and tissue damage, resulting in a septic shock with or without acute respiratory distress syndrome (ARDS) [29]. ARDS is responsible for approximately 70% of fatal COVID-19 cases [30]. ARDS may also occur from a variety of mechanisms, including the neutrophil activation and increased (micro)coagulation which is indicated by increased levels of CRP [31]. Vitamin D can reduce the CRP levels and hence negatively modulate the inflammatory cytokine storm caused by COVID-19 [2]. In the present study, CRP levels did not differ according to the vitamin D status of severe COVID-19 patients; however, other laboratory findings including the number of platelets, NLR, blood pH, PCT, lactate, urea, creatinine and LDH concentrations varied in the patients with vitamin D deficiency, suggesting that vitamin D status affects the inflammatory defense mechanisms against SARS-CoV-2.

Although the studies on various vaccines and drug treatments against SARS-CoV-2 are still ongoing, the CP therapy has also been used in severe or critically ill patients selected for the treatment. This specific method is a passive strategy to provide specific immunity that can be suitable not only for the COVID-19 but also for the treatment of many other diseases. Although the success rate is still being investigated, CP has been applied by physicians, especially in the critically ill patients who have a high risk of mortality [32]. The trials including the group of ICU patients with COVID-19 presented controversial data for the benefit from CP [33,34]. In one of these trials, 28% of 617 patients receiving invasive mechanical ventilation were successfully weaned from ventilation by CP therapy [35]. In a very recent trial, the administration of CP within 9 days after the onset of COVID-19 symptoms reduced the risk of disease progression leading to hospitalization [36]. In our study, CP was administered to 12.9% of 194 severe COVID-19 patients, and no significant difference was found according to the Vitamin D status.

Hemodiafiltration is a renal replacement technique combining diffusion and convection to enhance solute removal in a wide spectrum of molecular weights. The function of hemodiafiltration is the same as dialysis used in the chronic renal failure. However, the difference of this treatment is that it functions more slowly over a longer period of time. In some COVID-19 patients with end-stage renal disease and acute kidney injury, the solutes should be eliminated, and electrolyte disturbances and pH values need to be corrected together with the removal of the liquid load [37]. In our study, the hemodiafiltration was applied in 30.2% of cases with vitamin D deficiency, which was significantly higher than those with normal levels of vitamin D (5.3%).

Hemoperfusion is a method used to purify the blood and separate the substances. The hemoperfusion device contains an electrically neutral microporous resin that is an efficient blood purification method in the clearance of cytokine storm that occurred in the patients with sepsis and treated using resin-directed hemoadsorption in the ICU. It was hypothesized that this treatment modality is effective in treating critically ill patients with COVID-19 with ARDS and hyperinflammatory syndrome by removing inflammatory factors from the plasma [38]. In our study, 29.3% of cases with vitamin D deficiency and 14.5% of cases with normal vitamin D levels received hemoperfusion treatment, and the difference was significant.

## 5. Conclusions

This retrospective cross-sectional cohort study examined the association between vitamin D status and secondary infections in patients with severe COVID-19 admitted in the ICU of a tertiary-level hospital in Turkey. These COVID-19 patients with vitamin D deficiency may carry an increased risk of poor prognosis of disease indicated by the respiratory status, GCS and APACHE scores and hematologic findings due to elevation of secondary microbiological infections. Additionally, the treatment methods for COVID-19 patients may vary according to the vitamin D status in the ICU. In conclusion, the results of this study confirm the high prevalence of vitamin D deficiency in people with severe COVID-19 hospitalized in the ICU (60.8%), especially the elderly. The positive association between vitamin D deficiency and the morbidity and mortality of COVID-19 suggest that examining serum 25(OH)D levels could be considered in the clinical practice of COVID-19 cases. Moreover, vitamin D supplementation could be considered in patients with vitamin D deficiency and insufficiency.

## Figures and Tables

**Table 1:** Demographical features of COVID-19 patients compared according to the vitamin D status.

	Overall (*N* = 194)	Total 25(OH)D <20 ng/mL (*n* = 118)	Total 25(OH)D ≥20 ng/mL (*n* = 76)	*p* Value
Age (years), Mean ± SD	64.93 ± 14.63	67.02 ± 14.47	61.70 ± 14.38	0.013 *
Sex, *N* (%) Male Female	106 (54.6) 88 (45.4)	70 (59.3) 48 (40.7)	36 (47.4) 40 (52.6)	0.103
Comorbidities, *N* (%) Hypertension Diabetes Mellitus CAD Others	102 (52.6) 65 (33.5) 45 (23.2) 99 (52.1)	60 (50.8) 40 (33.9) 29 (24.6) 60 (52.6)	42 (55.3) 25 (32.9) 16 (21.1) 39 (51.3)	0.548 0.885 0.694 0.859

* *p* < 0.05 vs. Total 25(OH)D ≥ 20 ng/mL group. SD: standard deviation, CAD: coronary artery disease.

**Table 2:** Comparison of clinical and vital findings of COVID-19 patients according to the vitamin D status.

	Overall (*N* = 194)	Total 25(OH)D <20 ng/mL (*n* = 118)	Total 25(OH)D ≥20 ng/mL (*n* = 76)	*p* Value
SAP (mmHg)	117.56 ± 23.57 (120.5)	114.28 ± 25.66 (120)	122.64 ± 18.96 (125)	0.004 *
DAP (mmHg)	63.99 ± 12.14 (66.5)	62.63 ± 12.14 (64)	66.11 ± 11.9 (69)	0.002 *
Pulse (min)	90.86 ± 15.56 (90)	92.48 ± 17.23 (91)	88.34 ± 12.22 (87)	0.071
Temperature (°C)	36.77 ± 0.43 (36.7)	36.77 ± 0.5 (36.6)	36.77 ± 0.31 (36.7)	0.975
Respiratory rate (min)	15.52 ± 2.09 (16)	15.49 ± 2.16 (15)	15.57 ± 1.99 (16)	0.556
Respiratory status, *N* (%) Spontaneous Intubation	132 (68.0) 62 (32.0)	69 (58.5) 49 (41.5)	63 (82.9) 13 (17.1)	0.001 *
Intubation duration (day)	3.04 ± 3.41 (2)	3.14 ± 3.88 (1)	2.80 ± 1.95 (2)	0.208
GCS	13.72 ± 4.09 (15)	12.54 ± 4.35 (15)	15.14 ± 3.26 (15)	0.001 *
APACHE score	26.79 ± 13.92 (29)	30.57 ± 13.71 (31)	20.73 ± 12.05 (19)	0.001 *
SOFA score	13.13 ± 4.88 (14)	13.06 ± 4.85 (14)	13.26 ± 5.03 (14)	0.957

All values are given as mean ± standard deviation (median) except the respiratory status. * *p* < 0.05. SAP: Systolic blood pressure, DAP: Diastolic blood pressure, GCS: Glasgow coma scale, APACHE: The Acute Physiology and Chronic Health Evaluation, SOFA: Sequential Organ Failure Assessment, * *p* < 0.05 vs. Total 25(OH)D ≥ 20 ng/mL group.

**Table 3:** The laboratory findings of COVID-19 patients at the admission to ICU compared according to the vitamin D status.

	Overall (*N* = 194)	Total 25(OH)D <20 ng/mL (*n* = 118)	Total 25(OH)D ≥20 ng/mL (*n* = 76)	*p* Value
WBC (×10^3^)	15.62 ± 14.02 (12.5)	14.4 ± 12.65 (11.3)	17.5 ± 15.82 (13.3)	0.072
HGB (g/dL)	10.85 ± 2.93 (11.2)	11 ± 2.73 (11.3)	10.6 ± 3.87 (11.1)	0.655
PLT (×10^3^)	266,360.82 ± 105,520.69 (267,000)	246,500 ± 111,589.43 (252,000)	297,197.37 ± 87,426.92 (270,000)	0.002 *
Neutrophil (%)	13.14 ± 13.79 (9.7)	13.86 ± 16.14 (9.7)	12.03 ± 9 (9.7)	0.371
NLR	19.22 ± 16.8 (13.8)	20.97 ± 17.32 (16)	16.5 ± 15.7 (11.4)	0.015 *
PLR	50.88 ± 53.5 (35.7)	47.75 ± 49.24 (35.7)	55.74 ± 59.53 (34.7)	0.536
CRP (mg/L)	124.66 ± 87.96 (120.5)	122.49 ± 87.31 (113.7)	127.99 ± 89.44 (130.2)	0.687
PCT (ng/mL)	4.22 ± 11.34 (0.4)	4.52 ± 11.9 (0.5)	3.75 ± 10.46 (0.2)	0.020 *
Troponin (pg/mL)	818.34 ± 4015.79 (26.3)	689.52 ± 2967.82 (26.8)	1017.58 ± 5258.5 (22.6)	0.147
D-Dimer (ng/mL)	3139.58 ± 4770.11 (1461)	3468.01 ± 5437.86 (1623)	2635.99 ± 3483.64 (1214)	0.099
pH	7.4 ± 0.13 (7.4)	7.38 ± 0.15 (7.4)	7.43 ± 0.1 (7.5)	0.019 *
Lactate (mg/dL)	2.41 ± 2.59 (1.8)	2.71 ± 2.85 (1.9)	1.95 ± 2.05 (1.6)	0.019 *
AST (U/L)	105.6 ± 238.34 (41)	101.78 ± 207.24 (42)	111.53 ± 281.28 (39)	0.191
ALT (U/L)	69.92 ± 119.71 (35.5)	67.29 ± 93.76 (39)	74 ± 152.11 (31)	0.103
Urea (mg/dL)	67.45 ± 46.3 (55)	72.91 ± 47.18 (63)	58.99 ± 43.88 (43.5)	0.007 *
Creatinine (mg/dL)	1.32 ± 0.92 (1)	1.47 ± 1.02 (1.2)	1.09 ± 0.68 (0.8)	0.001 *
LDH (mg/dL)	592.97 ± 374.13 (498.5)	614.84 ± 338.46 (540.5)	559.01 ± 423.76 (455.5)	0.021 *
Ferritin (ng/mL)	779.71 ± 674.65 (522.3)	809.7 ± 649.05 (610.3)	732.33 ± 715.51 (474.9)	0.161
Fibrinogen	276.87 ± 132.16 (312.7)	310.73 ± 119.62 (322.4)	241.79 ± 137.4 (256.7)	0.224

All values are given as mean ± standard deviation (median). * *p* < 0.05. ICU: Intensive care unit, WBC: White blood cells, HGB: Hemoglobin, PLT: Platelets, NLR: Neutrophil/Lymphocyte ratio, PLR: Platelet/Lymphocyte Ratio, CRP: C-reactive protein, PCT: Procalcitonin, LDH: Lactate dehydrogenase, AST: Aspartate aminotransferase, ALT: Alanine aminotransferase.

**Table 4:** Comparison of treatments for COVID-19 patients according to the vitamin D status.

Treatment Method	Overall (*N* = 194)	Total 25(OH)D <20 ng/mL (*n* = 118)	Total 25(OH)D ≥20 ng/mL (*n* = 76)	*p* Value
CP	25 (12.9)	13 (11)	12 (15.8)	0.454
Hemodiafiltration	39 (20.3)	35 (30.2)	4 (5.3)	0.000 *
Hemoperfusion	45 (23.4)	34 (29.3)	11 (14.5)	0.028 *
Methylprednisolone	76 (39.2)	45 (38.1)	31 (40.8)	0.712
Actemra	61 (31.4)	39 (33.1)	22 (28.9)	0.548
Favipravir	184 (94.8)	111 (94.1)	73 (96.1)	0.743
Plaquenil	48 (24.7)	28 (23.7)	20 (26.3)	0.813

All values are given as *N* (%). * *p* < 0.05 CP: Convalescent (immune) plasma.

**Table 5:** Comparison of the prognosis of all COVID-19 patients according to the vitamin D status.

	Overall (*N* = 194)	Total 25(OH)D <20 ng/mL (*n* = 118)	Total 25(OH)D ≥20 ng/mL (*n* = 76)	*p* Value
Weaning Duration (day) Mean ± SD (Median)	8.16 ± 6.28 (7)	8.29 ± 6.04 (8)	7.90 ± 6.84 (6)	0.612
Hospitalization in ICU (day) Mean ± SD (Median)	10.53 ± 7.59 (9)	10.73 ± 7.41 (10)	10.21 ± 7.92 (8)	0.308
Mortality, *N* (%)	111 (57.2)	94 (79.7)	17 (22.4)	0.000 *

SD: Standard deviation * *p* < 0.05.

**Table 6:** Comparison of the microbiological findings of COVID-19 patients according to the vitamin D status.

		Overall (*N* = 194)	Total 25(OH)D <20 ng/mL (*n* = 118)	Total 25(OH)D ≥20 ng/mL (*n* = 76)	*p* Value
Growth in culture, *N* (%)		121 (62.4)	81 (68.6)	40 (52.6)	0.025 *
Microorganismal proliferation *N* (%)	Sensitive	61 (50.4)	41 (50.6)	20 (50)	0.035 *
	Moderately sensitive	7 (5.8)	1 (1.2)	6 (15)	
	Resistance	28 (23.1)	21 (25.9)	7 (17.5)	
	Sensitive/Resistance	15 (12.4)	10 (12.3)	5 (12.5)	
	Moderately Sensitive/Resistance	10 (8.3)	8 (9.9)	2 (5)	
Proliferation duration Mean ± SD (Median)		7.43 ± 4.54 (7)	7.64 ± 4.60 (8)	7.0 ± 4.43 (5.5)	0.441

SD: Standard deviation * *p* < 0.05.

**Table 7:** Comparison of the prognosis of COVID-19 patients who had microbiological growth in cultures compared according to the vitamin D status.

	Overall (*N* = 121)	Total 25(OH)D <20 ng/mL (*n* = 81)	Total 25(OH)D ≥20 ng/mL (*n* = 40)	*p* Value
Hospitalization in ICU (day) Mean ± SD (Median)	12.14 ± 8.45 (11)	12.21 ± 8.11 (7)	12.0 ± 9.21 (10)	0.368
Mortality, *N* (%)	89 (73.6)	74 (91.4)	15 (37.5)	0.000 *

SD: Standard deviation * *p* < 0.05.

## Data Availability

All data are available. The corresponding author will be given upon request.
